# Pharmacist-led deprescribing interventions for cancer patients in a specialist palliative care setting

**DOI:** 10.1007/s00520-025-09341-9

**Published:** 2025-03-26

**Authors:** Ciarán McAdam, Eimear O’Dwyer, Kieran Dalton

**Affiliations:** 1 Pharmacy Department, Our Lady’s Hospice & Care Services, Dublin, Ireland; 2 Pharmaceutical Care Research Group, School of Pharmacy, University College Cork, Cork, Ireland

**Keywords:** Cancer, Deprescribing, Drug therapy, Palliative Care, Pharmacist, Polypharmacy

## Abstract

**Purpose:**

This study aimed to determine the prevalence of potentially inappropriate medications (PIMs) among adult cancer patients in palliative care, the rate at which physicians implemented pharmacists’ deprescribing recommendations, and some cost implications of deprescribing.

**Methods:**

Medication reconciliation was performed for each eligible patient, with both the OncPal deprescribing guideline and clinical judgement applied to identify PIMs. PIM prevalence was evaluated for each medication class. The physician recommendation implementation rate and medication cost savings were calculated.

**Results:**

In the 48 included patients, 25.2% of medications were PIMs (mean 2.4/patient) - with 86.7% OncPal-defined PIMs, most commonly vitamins, medications for gastro-oesophageal reflux disease (GORD), and lipid-modifying agents. Pharmacist deprescribing recommendations were implemented 71.7% of the time, equivalent to 1.7 fewer medications per patient. The 28-day cost was €948.27 for deprescribed PIMs. Implementation rates varied based on patient admission type, with a significantly higher (*p*<0.05) rate in those admitted for end-of-life care (83.3%) versus symptom control (65.1%) and respite (30%) admissions. Recommendations to deprescribe GORD medications had a significantly lower rate of implementation (26.7%) compared to all other medications (*p*<0.0001).

**Conclusion:**

This study underscores the benefits of pharmacist-led deprescribing in inpatient palliative care, resulting in cost savings and reduced medication burden. There is a notable need for proactive deprescribing before reaching inpatient care. Different deprescribing rates across medication types highlight the significance of reviewing medications which may have a role in symptom management. The omission of some medications from OncPal emphasises the importance in refining future deprescribing guidelines in palliative care.

**Supplementary Information:**

The online version contains supplementary material available at 10.1007/s00520-025-09341-9.

## Introduction

When managing patients with palliative care needs, the overarching goal is to maximise comfort and quality of life (QoL). Medications play a critical role in symptom management in palliative care; however, as a patient’s disease progresses and life expectancy becomes more limited, certain medications may become inappropriate or unnecessary [1, 2]. Towards the end of life, the treatment focus may shift from curative or life-prolonging therapies to comfort and symptom control. Medications that were once prescribed for managing chronic conditions may no longer be aligned with palliative goals of care, as they are less likely to improve patients’ QoL [3]. However, whilst the evidence suggests that preventative medications should be discontinued or reduced in those with palliative care needs, many patients continue to receive such medications or those not specifically intended for symptom relief [1, 4, 5].

Medications may also propagate an increased risk of harm to patients with palliative care needs as they become frailer and more susceptible to potential adverse drug reactions (ADRs). Medications which were previously tolerated might now cause significant harm [6]. Physiological changes and co-morbidities can significantly alter drug pharmacokinetics in patients with limited life expectancy. In addition, certain conditions common in palliative care patients can lead to a progressive decline in liver and kidney function, which in turn has substantial implications for the effectiveness and safety of medications [7]. Owing to these shifts in pharmacokinetic and pharmacodynamic properties, patients receiving palliative care may face an elevated ADR risk [3, 7]. In addition to the inherent risks associated with medications in patients with palliative care needs, polypharmacy - most commonly defined as the use of ≥5 medications [3, 8, 9] - has been identified as a significant risk factor and predictor of ADRs for this patient group [6, 10], with it being prevalent in as many as three-quarters of all patients receiving palliative care [11]. Therefore, medication appropriateness warrants careful consideration in patients with palliative care needs to mitigate the risk of potential harm.

Potentially inappropriate medications (PIMs) are characterised as those for which the risk of adverse effects or complications outweighs the potential clinical benefits [3, 12]. Patients with terminal or life-limiting conditions frequently confront the detrimental effects of both PIMs and polypharmacy, consequences of which may include diminished QoL, ADRs, falls, hospitalisations, and even premature death [4, 9, 10, 13]. Studies have revealed that PIMs are frequently prescribed to cancer patients in palliative care, with PIM prevalence found to range anywhere from 12.8 to 95%, depending on the measure used [2, 3, 11, 14–22].

A method of reducing the number of PIMs prescribed for patients, and in turn minimising inappropriate polypharmacy, is deprescribing. Several studies have shown that deprescribing is effective in reducing the number of PIMs, drug interactions, and costs to both patients and healthcare systems, as well as improving patients’ QoL [1, 5, 6, 10, 23]. Determining whether a medication is inappropriate and therefore a candidate for deprescribing can be difficult; for this reason, several tools and guidelines have been developed to aid deprescribing in patients with palliative care needs [1, 3, 24, 25]. One such tool is the OncPal guideline [3], which provides evidence-based recommendations for safe and effective deprescribing in cancer patients with palliative care needs. The existing evidence base for deprescribing in palliative care (including the use of such tools) is heavily reliant on observational studies, with scant representation from interventional research employing these guidelines in clinical practice. It is essential to understand how these guidelines perform in the real world, including their effectiveness in identifying PIMs, the feasibility in their application, and acceptability to both patients and clinicians. Given the paucity of interventional research involving such tools in this area, the present study aimed to evaluate the impact of pharmacist-provided deprescribing recommendations (guided by OncPal) in minimising PIMs in an adult inpatient palliative care setting.

## Methods

### Study setting

Our Lady’s Hospice and Care Services (OLH&CS) provides inpatient palliative care to adult patients (i.e. aged ≥18 years only) with cancer and other life-limiting conditions across three locations in or near Dublin, Ireland (Harold’s Cross, Blackrock, and Wicklow), serving a population of >1 million people [26]. At the time of the study, the total bed capacity for the palliative care units (PCUs) in these locations was 36, 12, and 15 beds respectively, making it Ireland’s largest provider of specialist inpatient palliative care. A clinical pharmacy service was provided to each PCU as part of a consultant-led multi-disciplinary team. This service was performed at ward level and involved reviewing each patient’s medications, making any endorsements, or suggesting changes where appropriate. Pharmacists aimed to conduct medication reconciliation within 24 h of admission or the next working day, with this recorded in the patient’s drug chart, which also contained a comments section used to communicate any additional information about medications, such as whether they were identified as potentially inappropriate. Alternatively, information on any medication-related issues may be entered into the patient’s healthcare record by the pharmacist. The above procedures represented standard pharmaceutical care in the PCUs at the time of this study.

### Study design and reporting

This was a single-centre, prospective study that observed and evaluated the effects of pharmacist-led interventions aimed at deprescribing PIMs for cancer patients with palliative care needs around the medication reconciliation process. Based on resource constraints and the expected average number of eligible PCU admissions of seven patients per week, an initial sample size of 56 patients was targeted in an 8-week period.

The Template for Intervention Description and Replication (TIDieR) checklist [27] guided study conduct and reporting (Appendix 1).

### Eligibility criteria

Patients were deemed eligible for inclusion if they had a primary diagnosis of cancer, an estimated prognosis of <6 months (as per medical notes), aged ≥18 years at the time of admission, and prescribed ≥1 medication on PCU admission. Patients were excluded from the study if they were discharged before medication reconciliation was completed.

### Data collection

Prior to data collection, the lead study pharmacist met with all pharmacists who could potentially be involved in data collection (*n*=4). A copy of the OncPal guideline and data collection tool were provided to the pharmacists, all of whom were subsequently involved in the intervention. Data collection was performed over a 12-week period between 20^th^ March and 12^th^ June 2023. To test feasibility and identify any potential issues that would need to be addressed, a 1-week pilot of data collection was conducted by the lead study pharmacist from the 13^th^ of March 2023.

Patient data such as age, gender, primary diagnosis, route of admission, admission type, estimated prognosis, and total number of regular medications prescribed were collected. Details were also recorded for each PIM identified, such as the medication’s name, dose, frequency, method of communication used to intervene, and whether the deprescribing recommendation made was implemented. If a recommendation was not implemented, a reason for this was sought and recorded.

### Deprescribing intervention

The OncPal guideline describes, with explanations, when medications may be considered PIMs in palliative care, and encompasses aspirin, dyslipidaemia medications, antihypertensives, osteoporosis medications, oral hypoglycaemics, medications for peptic ulcer prophylaxis, vitamins, minerals, and complementary alternative medications [3].

After conducting medication reconciliation, OncPal criteria were applied by a pharmacist to a patient’s medications to identify PIMs. At this stage, during a comprehensive clinical assessment that considers the patient’s medications, medical history, current clinical conditions, and any other pertinent factors, a pharmacist had the discretion to identify and recommend the deprescribing of other PIMs outside of OncPal’s scope. Where a PIM was identified, a recommendation was made to the medical team to either reduce the dose or discontinue, with advice on tapering if necessary. It is standard practice in the hospital for pharmacists to initially provide recommendations to the medical team, so patients are only consulted after this.

The deprescribing recommendations were communicated verbally where possible, as this typically results in higher rates of acceptance [28]. Written communication alone was also deemed acceptable, as a recent study in OLH&CS found no statistically significant difference between the implementation rate of recommendations made verbally or in written form [29]. Written recommendations were entered into the aforementioned comments section in the patient’s drug chart or in the written healthcare record. The lead study pharmacist was the sole pharmacist that provided recommendations across all three locations (as other pharmacists were based at one location only).

### Data analysis

Descriptive statistics were performed using Microsoft® Excel and IBM® SPSS Statistics Version 28 for patient demographics and number of medications. The % prescriber implementation rates were calculated by dividing the number of pharmacist recommendations implemented over the total number made, and then multiplying this by 100. Chi-square tests and *t*-tests were utilised to assess the statistical differences among the data subgroups. A *p*-value of <0.05 was set as the threshold for statistical significance.

The total number of medication administrations was calculated from the dose and frequency data. The medication cost was calculated from national standard reimbursement prices [30], and did not include community pharmacist dispensing fees. The corrected unit price (i.e. cost per individual dose) was calculated and used to determine the PIM cost for 28-day and 1-year (i.e. 365 days) periods. Where a dose reduction occurred (e.g. pantoprazole 40mg to 20mg), the cost difference between the two medications was used as the cost saving from deprescribing.

## Results

### Patients

Forty-eight patients were included in this study. The mean age was 70 years (SD 12.8; range 42–97), with other patient characteristics displayed in Table 1. A full list of the patients’ primary diagnoses is in Appendix 2, with the two most frequently observed being malignant neoplasm of the brain (14.6%) and malignant neoplasm of the bronchus and lung (12.5%).

**Table 1:** Number of medications and PIMs according to patient characteristics

Patient characteristic	*n* (%)	Mean number of medications (SD)	Mean number of PIMs (SD)
**Gender**			
Female	27 (56.3%)	9.2 (3.5)	2.3 (1.7)
Male	21 (43.8%)	9.5 (1.5)	2.4 (1.5)
**Source of admission**			
Hospital	26 (54.2%)	10.5 (3.3)	2.1 (1.5)
Community palliative care teams	22 (45.8%)	8.0 (2.9)	2.6 (1.6)
**Admission type**			
End-of-life care	27 (56.3%)	9.7 (3.7)	2.2 (1.4)
Symptom control	18 (37.5%)	9.2 (3.1)	2.4 (1.6)
Respite	3 (6.3%)	7.7 (2.1)	3.3 (3.2)
**Prognosis on admission**			
<1 week	1 (2.1%)	3 (0)	1 (0)
1–4 weeks	25 (52.1%)	9.1 (3.6)	2.4 (1.7)
1–3 months	17 (35.4%)	10.1 (2.9)	2.2 (1.1)
>3 months	5 (10.4%)	9.2 (3.1)	3.2 (2.5)

*SD* standard deviation

### Medications

Patients were prescribed a mean of 9.4 regular medications (SD 3.4; range 3–16). Approximately one quarter (*n*=113; 25.2%) of medications recorded during medication reconciliation (*n*=449) were PIMs. All patients were deemed to have ≥1 PIM, with a mean of 2.4 per patient (SD 1.6; range 1–7).

Table 1 shows the mean number of medications and PIMs according to patient characteristics. Whilst the mean number of regular medications was significantly lower for those admitted from the Community Palliative Care Teams (CPCTs) versus hospital (8 versus 10.5; *p*=0.01), the mean number of PIMs was lower in patients coming from hospital (2.1 versus 2.6; *p*=0.26). Similarly, the mean number of regular medications was lower for those admitted for respite compared to end-of-life care and symptom control (7.7 versus 9.7 and 9.2; *p*=0.37 and *p*=0.44), whilst the mean number of PIMs was higher in patients admitted for respite compared to end-of-life care and symptom control (3.2 versus 1.4 and 1.6; *p*=0.27 and *p*=0.42).

Table 2 shows all medications identified as PIMs, ordered according to the Anatomical Therapeutic Chemical (ATC) classification main anatomical or pharmacological groups (1^st^ level). The three most common groups here were medications for the alimentary tract and metabolism (*n*=59; 52.2%), medications for the cardiovascular system (*n*=34; 30.1%), and medications for blood and blood forming organs (*n*=11; 9.7%). The three most common pharmacological subgroups (ATC 2^nd^ level) for PIMs identified were vitamins (26.6%), drugs used to manage gastro-oesophageal reflux disease (GORD) and peptic ulcer (13.3%), and lipid-modifying agents (11.5%). Most PIMs identified were OncPal-defined PIMs (*n*=98; 86.7%). Other PIMs not identified by OncPal (*n*=15; 13.3%) are denoted with superscripted letters in Table 2.

**Table 2:** Medications identified as PIMs and recommended for deprescribing

Medication (ATC Classification code)	PIMs, *n* (% total) Total: 113	PIMs deprescribed, *n* (% deprescribed) Total: 81
**Drugs for gastro-oesophageal reflux disease (A02)**	**15 (13.3%)**	**4 (4.9%)**
Pantoprazole (A02BC02)	6	2
Lansoprazole (A02BC03)	5	1
Esomeprazole (A02BC05)	2	0
Omeprazole (A02BC01)	1	1
Famotidine (A02BA03)	1	0
**Drugs for constipation (A06)**	**1 (0.9%)**	**1 (1.2%)**
Lactulose (A06AD11)^a^	1	1
**Drugs used in diabetes (A10)**	**3 (2.7%)**	**2 (2.5%)**
Gliclazide (A10BB09)	1	0
Glimepiride (A10BB12)	1	1
Sitagliptin (A10BH01)	1	1
**Vitamins (A11)**	**30 (26.5%)**	**27 (33.3%)**
Cholecalciferol (A11CC05)	11	11
Folic acid (A11CC01)	10	10
Multivitamin preparations (A11EA02)	5	3
Thiamine (A11DA01)	3	3
Vitamin E (A11HA03)	1	0
**Mineral supplements (A12)**	**10 (8.8%)**	**7 (8.6%)**
Calcium carbonate (A12AA04)	1	1
Calcium carbonate/cholecalciferol (A12AX)	8	6
Magnesium oxide (A12CC10)	1	0
**Antithrombotic agents (B01A)**	**9 (8.0%)**	**7 (8.6%)**
Aspirin (B01AC06)	4	3
Enoxaparin (B01AB05)^a^	3	3
Clopidogrel (B01AC04)^a^	1	1
Apixaban (B01AF02)^a^	1	0
**Iron preparations (B03A)**	**2 (1.8%)**	**2 (2.5%)**
Ferrous fumarate (B03AA02)	2	2
**Antiarrhythmics, class III (C01BD)**	**1 (0.9%)**	**0 (0%)**
Amiodarone (C01BD01)^a^	1	0
**Diuretics (C03)**	**2 (1.8%)**	**0 (0%)**
Furosemide (C03CA01)	1	0
Spironolactone (C03CA01)^b^	1	0
**Beta-blocking agents (C07)**	**5 (4.4%)**	**5 (6.2%)**
Bisoprolol (C07AB07)	5	5
**Calcium channel blockers (C08)**	**7 (6.2%)**	**4 (4.9%)**
Lercanidipine (C08CA13)	4	4
Amlodipine (C08CA01)	3	0
**Angiotensin-converting-enzyme inhibitors and combinations (C09A, C09B)**	**3 (2.7%)**	**3 (3.7%)**
Ramipril (C09AA05)	2	2
Ramipril/felodipine (C09BB05)	1	1
**Angiotensin II receptor blockers and combinations (C09C, C09D)**	**3 (2.7%)**	**1 (1.2%)**
Candesartan (C09CA06)	1	0
Telmisartan/hydrochlorothiazide (C09DA07)	1	1
Olmesartan/amlodipine (C09DB02)	1	0
**Lipid-modifying agents (C10)**	**13 (11.5%)**	**11 (13.6%)**
Atorvastatin (C10AA05)	9	8
Ezetimibe (C10AX09)	3	2
Rosuvastatin (C10AA07)	1	1
**Drugs for urinary frequency and incontinence (G04BD)**	**2 (1.8%)**	**2 (2.5%)**
Solifenacin (G04BD08)^a^	1	1
Mirabegron (G04BD12)^a^	1	1
**Alpha-adrenoreceptor antagonists (G04C)**	**2 (1.8%)**	**2 (2.5%)**
Tamsulosin (G04CA02)^a^	2	2
**Hormone antagonists and related agents (L02B)**	**1 (0.9%)**	**1 (1.2%)**
Degarelix (L02BX02)^a^	1	1
**Anti-inflammatory and antirheumatic products (M01)**	**1 (0.9%)**	**0 (0%)**
Glucosamine (M01AX05)	1	0
**Bisphosphonates (M05BA)**	**1 (0.9%)**	**1 (1.2%)**
Alendronic acid (M05BA08)	1	1
**Antipsychotics (N05A)**	**1 (0.9%)**	**1 (1.2%)**
Prochlorperazine (N05AB04)^a^	1	1
**Antihistamines for systemic use (R06A)**	**1 (0.9%)**	**0 (0%)**
Desloratadine (R06AX27)^a^	1	0

*ATC* Anatomical Therapeutic Chemical, *PIM* Potentially inappropriate medication

^a^Denotes medications not included within the OncPal deprescribing guideline

^b^Included within OncPal but not as indicated for the patient

### Medication administrations associated with deprescribing

The total number of individual medication administrations for PIMs identified amounted to 3518 administrations per 28-day period, equivalent to a mean 2.6 administrations per patient per day. Deprescribing recommendations which were implemented resulted in a reduction of 2314 individual medication administrations per 28-day period, equivalent to a mean reduction of 1.7 administrations per patient per day.

### Implementation of deprescribing recommendations

Pharmacists made 113 deprescribing recommendations (*n*=99 for discontinuation and *n*=14 for dose reduction), of which 71.7% (*n*=81) were implemented by physicians. For 30/48 patients (62.5%), all deprescribing recommendations were implemented. The mean number of medications prior to the deprescribing recommendations was 9.4, which reduced significantly to 7.7 (*p*=0.01) following implementation (i.e. 1.7 fewer medications per patient). These deprescribing recommendations were communicated verbally with a written record of the recommendation (*n*=79; 69.9%) or only in written form with no verbal communication (*n*=34; 30.1%). The implementation rates of verbal recommendations and written-only recommendations were 72.2% and 70.6% respectively (*p*>0.05). Deprescribing recommendations for OncPal-defined PIMs were implemented 71.4% of the time (70/98). Similarly, 73.3% (11/15) of recommendations to deprescribe other PIMs were implemented.

Recommendations to deprescribe PIMs were implemented for end-of-life care, symptom control, and respite admissions at rates of 83.3%, 65.1%, and 30% respectively. The most significant difference was seen between end-of-life care and respite admissions (*p*<0.001), followed by end-of-life care and symptom control admissions (*p*=0.03), and respite and symptom control admissions (*p*=0.04).

From the 28.3% (*n*=32) of deprescribing recommendations which were not implemented during the study, the pharmacological subgroup most frequently implicated were medications to manage peptic ulcer and GORD, accounting for nearly one-third of these (*n*=11; 34.4%). Deprescribing recommendations for these medications had a significantly lower implementation rate compared to that of all other PIM recommendations combined (26.7% versus 78.6%; *p*<0.0001).

Whilst physicians indicated that none of the pharmacists’ recommendations were inappropriate based on information available to the pharmacists, the reasons recorded for non-implementation of recommendations are shown in Table 3.

**Table 3:** Reasons for non-implementation of deprescribing recommendations

Reason for not deprescribing (including number of patients and medications involved)	Frequency
Patient’s preference for the medication to continue (*n*=4 patients). This pertained to two multivitamins, and one each of: vitamin E, glucosamine, calcium carbonate/cholecalciferol, ezetimibe, a PPI, glucosamine, and amlodipine.	7
New observations made or information provided during the admission precluded deprescribing: - four antihypertensives not deprescribed due to high blood pressure readings (*n*=3 patients) - undocumented indications: one PPI and calcium carbonate/cholecalciferol not deprescribed due to undocumented patient-reported reflux and hypocalcaemia (*n*=1 patient each).	6
The admission was planned to be short and deal with specific issues, so changes to other medications were not required (*n*=1 patient).	6
Medical team wanted to review/monitor patient's condition, and it was agreed that deprescribing could be considered later (*n*=3 patients). This pertained to two PPIs, and one each of desloratadine, amlodipine, and aspirin.	5
Indication unclear and/or did not want to deprescribe in case it resulted in additional symptoms (*n=*4 patients), all of which pertained to PPIs.	4
It was planned to start other medications which would preclude deprescribing (*n*=2 patients). Both related to deprescribing PPIs when planning to start a non-steroidal anti-inflammatory drug or dexamethasone.	2
Prescriber preferred to continue for unclear reasons (*n*=2 patients), pertaining to amiodarone and a magnesium supplement.	2

*PPI* proton pump inhibitor. All recommendations provided were deemed safe by the medical team, based on the information available to pharmacists at the time of provision

### Medication costs associated with deprescribing

Based on reimbursement prices alone, the 28-day medication cost savings from discontinuation or dose reduction were calculated to be:

- €1,128.73 (€23.51 per patient) for all PIMs identified, corresponding to €306.54 per patient per annum.
- €948.27 (€19.76 per patient) for deprescribed PIMs, corresponding to €257.59 per patient per annum.

## Discussion

Deprescribing is gaining traction as a crucial element of patient-centred care, especially relevant in palliative care, where the focus shifts from active treatment to comfort, QoL, and symptom management. This study has helped address an important literature gap, in showing the success of pharmacist application of deprescribing guidelines as a prospective intervention within an inpatient palliative care setting. Nearly three quarters of pharmacists’ deprescribing recommendations were implemented (71.7%) within the study timeframe. This falls within the range of previous pharmacist interventions, where acceptance/implementation rates have varied, for example, between 54.8% and 93.9% [20, 31–33]. This study’s implementation rate was not consistent across all medication classes. Proton pump inhibitors (PPIs) and H_2_ receptor antagonists were deprescribed only 26.7% of the time, significantly lower (*p*<0.0001) than the 78.6% for all other medications. Deprescribing being less successful for these classes has been observed in previous palliative care deprescribing studies [18, 22]. One possible reason for this lower rate is that unlike other PIM categories, PPIs and H_2_ receptor antagonists can be used for symptom management and to improve patients’ QoL, i.e. having some utility towards the goal of improving symptoms. Consequently, and as indicated by some of this study’s physicians’ reasons for not deprescribing, there may be some reluctance to discontinue these medications due to the possibility of adverse drug withdrawal effects. Despite this reluctance, the labelling of PPIs as being potentially inappropriate is well-founded [3, 24]. They have been associated with *Clostridioides difficile* infection, bone loss, and fracture [34], and they are frequently identified as one of the most common PIMs in palliative care [1, 11, 15]. This shows the need for a clear risk-benefit evaluation to be conducted when considering PPI deprescribing in palliative care.

This study’s medication classes most frequently deemed potentially inappropriate included lipid-lowering agents, medications for gastrointestinal ulcer prophylaxis, and vitamins, which aligns with several other studies with a similarly high prevalence of these as PIMs [16–19]. This recurring pattern underscores the importance of more proactive medication review in palliative care. Patient preferences to continue medications, including these commonly identified PIMs, were established as the most common reason for not implementing deprescribing in this study; if these were acquired before recommendation provision, the implementation rate would have been 76.4% (81/106). Recognising and respecting these preferences is integral to person-centred care, especially in palliative setting where the overarching goal is to maximise QoL [35].

In an era where there is competing demand for the efficient use of resources, an area that offers significant potential for improvement is medication administration. As a result of deprescribing in this study, there were 2314 fewer individual medication administrations per 28-day period, a reduction of 1.7 administrations per patient per day. Studies which have timed nurses during medication administration have found that it takes 41–176 s to administer a single medication dose [36, 37]. Applying this to the main 36-patient unit in OLH&CS would translate to a saving of about 41.8–179.5 min of medication administration time per day. This saving could allow nursing staff to allocate more time to patient care, education, communication with families, and other vital tasks, thereby potentially enhancing the overall quality of care [38]. There seem to be no studies which have timed medication administration in palliative care settings; given that these times can vary depending on the severity of the patient’s condition [37], this may represent an avenue for future research.

The OncPal guideline was effective at identifying most of this study’s PIMs (86.7%), but not all, emphasising the value of a pharmacist’s clinical judgement. The most common PIM group identified independently of OncPal in this study were antithrombotics. Whilst some patients may be at higher risk of thrombosis and have appropriate indications for antithrombotics (including cancer-associated thrombosis) [39], the rationale for antithrombotics as PIMs would be that venous thromboembolism occurrence in cancer patients receiving palliative care is relatively rare, whilst the bleeding risk for some individuals is significant [18, 40]. The fact that these antithrombotics were identified as PIMs independent of OncPal underscores the need for pharmacists to exercise their judgement and be guided by broader clinical considerations. A previous study using OncPal similarly identified prophylactic anticoagulants as PIMs and suggested they should be considered for addition to the guideline [18]. Notably since study completion, a tool for deprescribing antithrombotics in palliative cancer patients has been published, which warrants further consideration [41].

The present study found that patients transferred from hospitals to the inpatient PCU were prescribed more medications on average compared to those admitted from CPCTs. From this, one might also expect that those admitted from hospital would have a higher average number of PIMs prescribed; however, the reverse was the case. These results seem to concur with previous studies which found a higher PIM prevalence in primary care [19] compared to hospital and hospice settings [11, 16–18, 20, 22, 23]. Possible explanations for this are that hospitalised patients may have more recent treatments and interventions whilst in hospital, or that their medical histories might be more acute or complex, thus requiring a broader range of medications [42]. For patients admitted from community settings, they may not have experienced such rigorous medical oversight, leading to fewer prescribed medications, but also a higher likelihood of PIMs due to the lower frequency of medication reviews. Within a hospital setting, patients often have their medications regularly reviewed by specialists, including pharmacists, which can significantly reduce PIM prescription [20, 33]. Therefore, an opportunity exists for more proactive deprescribing in the community before reaching the inpatient palliative care setting.

Deprescribing recommendations were communicated by two routes in this study: verbally or in writing, with similar implementation rates of 72.2% and 70.6% respectively. These findings align with a previous study conducted in the same institution, where negligible differences were found between a pharmacist’s verbal and written recommendation implementation rates by medical teams [29]. This reassuringly suggests that the mode of communication is not the primary determinant of implementation. Instead, other factors, such as recommendation complexity and trust-based collaborative relationships between pharmacists and physicians [43, 44], might play more pivotal roles. The present study’s findings become especially pertinent considering the development of electronic communications and medication management systems where digitised-written communications are key features [45]. Future deprescribing studies in this realm should consider designing their interventions using implementation science to enhance the implementation of such recommendations and sustainment of interventions [46].

### Strengths and limitations

A study strength was the attainment of physicians’ reasons for non-implementation of recommendations. However, patient preferences for non-implementation were not explored in great depth. Furthermore, due to resource constraints, this study has a small sample size and did not examine how deprescribing affected patients’ QOL, overall condition, or symptom management. Larger studies are warranted, especially to facilitate showing more potential differences for recommendations based on the type of medication, patient admission, and prognosis.

This study focused solely on deprescribing PIMs; similar future studies may consider a more holistic approach in also addressing potential prescribing omissions that may improve patients’ QoL or symptom management [24].

Multiple pharmacists were involved in this study across three PCUs, which aids the findings’ transferability. It was conducted in a setting that included patients admitted from both hospital and community, which allowed for a broad coverage of the palliative patient population. However, it exclusively focused on patients diagnosed with cancer; whilst such patients represent the majority of admissions to specialised inpatient PCUs in Ireland [47], similar future studies should explore the implications for those in the community setting and with non-cancer diagnoses to ensure a comprehensive understanding of deprescribing needs across the palliative care spectrum.

## Conclusion

This study has highlighted the value of pharmacist-led deprescribing within inpatient palliative care, showing a significantly reduced medication burden for patients and nursing staff, alongside considerable medication cost savings. The higher PIM prevalence among patients admitted from primary care suggests a need for more proactive deprescribing before reaching inpatient palliative care settings. Furthermore, the differing rates of deprescribing implementation across medication classes in this study emphasises the need for clarity on establishing indications for medications and which ones play a role in symptom management in palliative care, especially for PPIs.

The identification of PIMs outside of the OncPal guideline (e.g. antithrombotics) highlights the need for guideline refinement and the added value of clinical judgement as part of such interventions to address additional PIMs. Lastly, this study has indicated the role that patient preference plays in deprescribing in palliative care. Future studies should aim to evaluate QoL alongside such deprescribing, to ensure that medication management in palliative care is truly patient-centred, enhancing patients’ comfort and overall well-being.

## Supplementary Information

Below is the link to the electronic supplementary material.

Supplementary file1 (DOCX 33 KB)

## Data Availability

The data associated with this study will not be made publicly available.
