# Supplementary material for: Pharmacist-led deprescribing interventions for cancer patients in a specialist palliative care setting
Source: Support Care Cancer. 2025 Mar 26;33(4):321. doi: 10.1007/s00520-025-09341-9 (PMC11946936; doi:10.1007/s00520-025-09341-9)
Supplement: Supplementary file 1 — Supplementary file1 (DOCX 33 KB) [file 520_2025_9341_MOESM1_ESM.docx]

# Supplementary Information

Journal: Supportive Care in Cancer

Ciarán McAdam ^1^, Eimear O’Dwyer ^1^, Kieran Dalton ^2^.

^1^ Pharmacy Department, Our Lady's Hospice & Care Services, Dublin, Ireland.

^2^ Pharmaceutical Care Research Group, School of Pharmacy, University College Cork, Cork, Ireland.

Corresponding author: Kieran Dalton (ORCID iD: 0000-0003-1209-8079)

Email: kieran.dalton@ucc.ie

.

## Appendix 1: The TIDieR (Template for Intervention Description and Replication) Checklist

| **N° item** | **Item** | **Where located (Section)** |
| --- | --- | --- |
|  | **BRIEF NAME** |  |
| **1.** | Provide the name or a phrase that describes the intervention. | Title, Introduction, and Deprescribing intervention |
|  | **WHY** |  |
| **2.** | Describe any rationale, theory, or goal of the elements essential to the intervention. | Introduction (aim) |
|  | **WHAT** |  |
| **3.** | Materials: Describe any physical or informational materials used in the intervention, including those provided to participants or used in intervention delivery or in training of intervention providers | Data Collection |
| **4.** | Procedures: Describe each of the procedures, activites and.or processes used in the intervention, including any enabling or supportive activities | Deprescribing intervention |
|  | **WHO PROVIDED** |  |
| **5.** | For each category of intervention provider (e.g. psychologist, nursing assistant), describe their expertise, background and any specific training given. | Data Collection |
|  | **HOW** |  |
| **6.** | Describe the modes of delivery (e.g. face-to-face or by some other mechanism, such as internet or telephone) of the intervention and whether it was provided individually or in a group. | Deprescribing intervention |
|  | **WHERE** |  |
| **7.** | Describe the type(s) of location(s) where the intervention occurred, including any necessary infrastructure or relevant features. | Study setting |
|  | **WHEN and HOW MUCH** |  |
| **8.** | Describe the number of times the intervention was delivered and over what period of time including the number of sessions, their schedule, and their duration, intensity or dose. | Data Collection |
|  | **TAILORING** |  |
| **9.** | If the intervention was planned to be personalised, titrated or adapted, then describe what, why, when, and how. | Deprescribing intervention |
|  | **MODIFICATIONS** |  |
| **10.^ǂ^** | If the intervention was modified during the course of the study, describe the changes (what, why, when, and how). | Not applicable. |
|  | **HOW WELL** |  |
| **11.** | Planned: If intervention adherence or fidelity was assessed, describe how and by whom, and if any strategies were used to maintain or improve fidelity, describe them. | Not applicable. |
| **12.** | Actual: If intervention adherence or fidelity was assessed, describe the extent to which the intervention was delivered as planned. | Not applicable. |

**Appendix 2: Patients’ primary diagnoses**

| Frequency of diagnosis (ICD-10 code) | *n* (%) |
| --- | --- |
| Malignant neoplasm of brain (C71) | 7 (14.6%) |
| Malignant neoplasm of bronchus and lung (C34) | 6 (12.5%) |
| Malignant neoplasm of stomach (C16) | 4 (8.3%) |
| Malignant neoplasm of prostate (C61) | 4 (8.3%) |
| Malignant neoplasm of colon (C18) | 3 (6.3%) |
| Malignant neoplasms of breast (C50) | 3 (6.3%) |
| Malignant neoplasm of oesophagus (C15) | 2 (4.2%) |
| Malignant neoplasm of cervix uteri (C53) | 2 (4.2%) |
| Malignant neoplasm of corpus uteri (C54) | 2 (4.2%) |
| Malignant neoplasm of bladder (C67) | 2 (4.2%) |
| Carcinoma in situ of colon (D01) | 2 (4.2%) |
| Malignant neoplasm of rectum (C20) | 1 (2.1%) |
| Malignant neoplasm of gallbladder (C23) | 1 (2.1%) |
| Malignant neoplasm of accessory sinuses (C31) | 1 (2.1%) |
| Malignant neoplasm of larynx (C32) | 1 (2.1%) |
| Malignant neoplasm of ovary (C56) | 1 (2.1%) |
| Malignant neoplasm of kidney, except renal pelvis (C64) | 1 (2.1%) |
| Malignant neoplasm of other endocrine glands and related structures (C75) | 1 (2.1%) |
| Non-follicular lymphoma (C83) | 1 (2.1%) |
| Multiple myeloma and malignant plasma cell neoplasms (C90) | 1 (2.1%) |
| Benign neoplasm of mouth and pharynx (D10) | 1 (2.1%) |
| Benign neoplasm of other and ill-defined parts of digestive system (D13) | 1 (2.1%) |
